# The Effect of Financial Toxicity on the Association Between Perceived Social Support and Negative Emotions in Post‐Surgical Lung Cancer Patients: A Cross‐Sectional Study From High‐Middle Income Region in China

**DOI:** 10.1002/cam4.71256

**Published:** 2025-11-01

**Authors:** Julan Xiao, Lili Liu, Yanheng Xie, Sijiao Cheng, Binghu Lin, Lansong Qin, Fengjiao Chen, Huai An, Wanling Xu, Yi Wen, Weixiang Luo

**Affiliations:** ^1^ Department of Thoracic Surgery Shenzhen People's Hospital (The Second Clinical Medical College, Jinan University; The First Affiliated Hospital, Southern University of Science and Technology) Shenzhen Guangdong China; ^2^ School of Physical Education Yunnan Minzu University Yunnan China; ^3^ Department of Nursing Department of Nursing, Shenzhen People's Hospital (The Second Clinical Medical College, Jinan University; The First Affiliated Hospital, Southern University of Science and Technology) Guangdong China

**Keywords:** financial toxicity, lung cancer, negative emotions, perceived social support, post‐surgical

## Abstract

**Objective:**

To investigate the mediating role of financial toxicity in the relationship between perceived social support and negative emotions among post‐surgical lung cancer patients in China.

**Methods:**

A total of 447 lung cancer patients from China were recruited, participants completed the Comprehensive Scores for Financial Toxicity (COST), the Negative Emotion Screening Scale (NESS), and the Perceived Social Support Scale (PSSS). The hypothesized relations were explored using structural equation modeling via the bootstrapping approach. The study methods were compliant with the STROBE checklist.

**Results:**

Nearly half (42.51%, *n* = 190) of the participants experienced financial toxicity. Negative emotions were negatively associated with perceived social support (*r* = −0.13) and negatively associated with financial toxicity scores (*r* = −0.50) (Note: COST is a reverse scoring scale, lower COST scores illustrate severe financial toxicity). Perceived social support was positively correlated with financial toxicity scores (*r* = 0.26). Financial toxicity scores negatively predicted negative emotions (*β* = −0.504). Social support and financial toxicity could explain 25.3% of the variance in patients' negative emotions. Social support positively predicted financial toxicity (*β* = 0.257) and explained 6.60% of the variance in financial toxicity. Furthermore, financial toxicity completely mediated the association between perceived social support and negative emotions (*b* = −0.124; 95% confidence interval: −0.182, −0.086).

**Conclusion:**

The high prevalence of financial toxicity demonstrates its crucial role in patient outcomes. Our findings reveal that social support indirectly influences negative emotions through financial toxicity, establishing a clear pathway linking psychosocial and financial aspects of cancer care. These results suggest that integrated interventions targeting both financial burden and social support networks may be particularly effective in improving psychological outcomes among lung cancer patients in China's high‐middle income regions. Future healthcare policies should prioritize comprehensive support systems that address this complex interplay of socioeconomic and psychological factors.

## 1 Introduction

Lung cancer remains a formidable global health challenge, ranking as the second most prevalent malignancy and the primary cause of cancer‐associated mortality worldwide [1]. In China, this malignancy has emerged as the predominant cancer type, exhibiting both the highest incidence and mortality rates among all malignancies [2]. The Global Cancer Statistics 2022, published by the International Agency for Research on Cancer (IARC), documented an estimated 2.383 million new lung cancer diagnoses and 1.271 million associated fatalities globally [1]. Within China's healthcare landscape, the burden of lung cancer reached unprecedented levels in 2022, with 1.060 million newly diagnosed cases constituting 22.0% of all malignant neoplasms [3]. The disease culminated in 733,300 deaths, accounting for 28.5% of all cancer‐related mortality in the nation, thereby representing a critical public health challenge and imposing a substantial socioeconomic burden [3]. Survival metrics paint a particularly concerning picture: during 2012–2015, the lung cancer survival rate in China stood at merely 16.8% for males and 25.1% for females [4]. Even in nations with advanced healthcare systems, prognosis remains poor, with five‐year survival rates of 13% and 19% in the United Kingdom and United States, respectively [5]. In light of these sobering statistics, recent research paradigms have expanded to encompass the psychological dimensions of patient care [6].

Patients with lung cancer frequently experience significant psychological distress, primarily attributed to the disease's high mortality rate, substantial risk of recurrence, and prolonged therapeutic interventions [7]. These persistent and recurring stressors often precipitate adverse psychological manifestations [8]. Epidemiological studies have revealed that 15%–44% of lung cancer patients suffer from severe depression and anxiety, which lead to therapeutic resistance and poor treatment compliance [9].

With the advancement and widespread adoption of minimally invasive techniques for lung cancer treatment, surgical treatment remains the primary and most effective therapeutic approach for managing the disease [10]. However, the high‐risk nature of surgery, combined with surgical trauma and anesthesia, often exerts significant psychological and physiological impacts on patients. Studies have shown that lung cancer patients, compared to those with other malignancies, bear a disproportionately heavy symptom burden stemming from both the disease itself and its treatment, adversely affecting their quality of life and functional status throughout the disease trajectory [11]. During the postoperative recovery phase, the prolonged treatment duration, poor prognosis, and substantial financial burden further exacerbate symptoms and negative emotions, directly impairing patients' quality of life [12]. While lung cancer remains incurable at present, current treatments focus on disease control and life extension through pharmacological and therapeutic interventions [13]. Consequently, rising medical costs have become inevitable. Economic forecasting models project that the cumulative global economic impact of cancer therapeutics from 2020 to 2050 will amount to $25.2 trillion, with lung cancer representing the most substantial financial burden among all malignancies, projected to incur losses of $3.9 trillion [14]. The continuous evolution of pharmacotherapeutic options has resulted in an exponential increase in oncologic care costs, with average expenditures rising approximately tenfold over the past 15 years [15]. Additionally, the increasing adoption of advanced surgical technologies, including video‐assisted thoracoscopic surgery (VATS) and robotic‐assisted techniques, has been associated with escalating hospital expenditures [16].

The concept of “Financial Toxicity” (FT) has been widely adopted to encompass the multifaceted economic ramifications experienced by cancer patients undergoing continuous treatment, including various manifestations of financial hardship, burden, distress, stress, and economic strain [17]. The escalating financial burden on households has emerged as a significant threat to both survivors and their caregivers, potentially impeding reforms in public healthcare systems. This burden is primarily attributed to the substantial costs associated with cancer‐related diagnostics and treatments, the implementation of multiple and extended therapeutic regimens, and increased survival rates among cancer patients [18]. Notably, individuals diagnosed with lung cancer face an alarming risk of bankruptcy, with rates approximately four times higher than those without cancer [19]. These devastating financial consequences not only compromise patients' health‐related quality of life and psychological well‐being but may also significantly impact their long‐term oncological outcomes [16]. Such impacts manifest through increased symptom burden, diminished treatment compliance leading to poorer outcomes, and elevated levels of negative emotions and psychological distress [13]. The interplay between FT and psychological status among cancer patients has been associated with reduced treatment adherence and increased cancer‐specific mortality [20]. While research into FT continues to expand, current investigations have predominantly focused on identifying its determinants and examining its effects on patient outcomes [21]. The implementation of early screening protocols for both FT and mental health challenges could facilitate timely multi‐component interventions, ultimately enhancing the quality of life for cancer patients.

Studies have demonstrated that FT significantly compromises patients' quality of life by adversely affecting their physical, social, and emotional functioning [22]. Conversely, robust social support has been consistently associated with enhanced physical and psychological outcomes [23]. However, there remains a notable paucity of correlational research examining the interrelationships between perceived social support, FT, and negative emotional states specifically among cancer patients. This gap in the literature warrants further investigation. The present study aims to examine the complex interplay between perceived social support, FT, and negative emotions among post‐surgical lung cancer patients in China. We propose three hypotheses: first, we hypothesized that surgically treated lung cancer patients in China experience significant levels of FT. Second, we hypothesized that higher levels of perceived social support are inversely associated with negative emotions. Finally, we hypothesized that FT mediates the relationship between social support and negative emotional states.

## 2 Methods

### 2.1 Study Design, Setting, and Participants

A cross‐sectional observational study was conducted at Shenzhen People's Hospital, a tertiary public healthcare institution in Southern China, from September 2021 to May 2023. The study initially enrolled 470 participants who met the following inclusion criteria: (1) clinicopathologically confirmed diagnosis of lung cancer; (2) age ≥ 18 years; (3) underwent surgical intervention at the study institution; (4) demonstrated adequate cognitive capacity for reading comprehension and verbal expression; (5) and patients who agreed to voluntary participation and signed an informed consent in this study. Exclusion criteria were included: (1) patients who had concomitant physical diseases, such as illiteracy, inability to understand, and respond effectively to the study survey; (2) voluntary withdrawal from the study; (3) and/or pre‐existing psychiatric disorders or cognitive impairment.

Of the 470 eligible patients, thirteen declined participation citing privacy concerns or lack of interest, and ten were excluded due to invalid questionnaire completion (incomplete essential information or obvious self‐contradiction). After eliminating the ineffective data, 447 sets of data were finally used for statistical analysis (effective response rate = 95.1%).

### 2.2 Procedures

This investigation was conducted in Shenzhen, one of China's premier high‐income metropolitan centers. All research personnel underwent standardized training in questionnaire administration and were instructed to communicate both the study objectives and confidentiality protocols to participants. After obtaining lung cancer patients written consent to the survey, the requirements of filling in the questionnaires were distributed to the inpatients with lung cancer. In order to test the feasibility and suitability of the questionnaires, a pilot test including 20 lung cancer patients was conducted at an inpatient thoracic surgery department. The patients who participated were also asked for advice on questionnaire modifications. Pilot test data were not used for the final statistics. The trained nurses distributed the final version of the questionnaires to the lung cancer patients for data collection.

### 2.3 Measures

#### 2.3.1 Independent Variable: Perceived Social Support

The Perceived Social Support Scale (PSSS) was utilized to assess the social support perceived by lung cancer patients. Originally developed by Zimet et al. [24] and subsequently adapted for the Chinese population by Jiang, the scale comprises three dimensions: family support, friend support, and support from significant others. The PSSS is a 12‐item self‐reported instrument, with each item rated on a 7‐point Likert scale ranging from 1 (strongly disagree) to 7 (strongly agree). The total score of the scale ranges from 12 to 84, with higher scores reflecting greater perceived social support. In this study, the PSSS demonstrated excellent internal consistency, with a Cronbach's alpha coefficient of 0.949.

#### 2.3.2 Mediator: Financial Toxicity

Financial toxicity was assessed using the Comprehensive Score for Financial Toxicity‐Patient‐Reported Outcome Measure (COST‐PROM), an instrument originally developed and validated by de Souza et al. [25]. The scale was subsequently translated, culturally adapted, and validated for the Chinese population by Yu et al. [26], demonstrating robust psychometric properties. The COST‐PROM consists of 11 items encompassing two dimensions: positive financial well‐being and negative psychosocial responses. Patients were asked to respond using a 5‐point Likert scale ranging from 0 (not at all) to 4 (very much). The total score ranges from 0 to 44, with lower scores indicating higher levels of FT and worse financial conditions. In the present study, the COST‐PROM exhibited strong internal consistency, with a Cronbach's alpha coefficient of 0.882.

#### 2.3.3 Dependent Variable: Negative Emotions

Negative emotions were assessed using the Chinese version of the Negative Emotions Screening Scale for Inpatients (NESSI), an instrument specifically developed within the Chinese cultural context [27]. The NESSI encompasses four distinct dimensions: illness‐related fear, indignation, somatization, and depression. This 17‐item self‐reported instrument employs a 5‐point Likert scale, with responses ranging from 1 (none) to 5 (always). The total score of the scale ranges from 17 to 85, with higher scores reflecting higher levels of negative emotions. The psychometric properties of the NESSI have been extensively validated in previous studies among Chinese populations, demonstrating robust internal consistency with Cronbach's alpha coefficients ranging from 0.762 to 0.925 [27]. Cronbach's alpha in the current study was 0.927.

#### 2.3.4 Covariates: Sociodemographic and Clinical Information

Sociodemographic data collected from lung cancer patients included age, gender, place of residence, employment status, financial burden of medical expenses, impact of the lung cancer diagnosis on retirement age, marital status, monthly household income, educational attainment, medical insurance coverage, disease‐related knowledge, and smoking history. Clinical information gathered encompassed tumor stage, pathological subtype, history of chemotherapy, and duration of cancer (in years).

### 2.4 Statistical Analysis

Categorical data were summarized using frequencies and percentages. Continuous data were expressed with mean and standard deviation or median (quartile). We conducted tertiles of COST scores and defined high FT as the bottom tertile and low FT as the top two tertiles; data were stratified by high and low FT [28]. The differences between groups were analyzed using the Student's *t*‐test, one‐way analysis of variance, Mann–Whitney *U* test, or Kruskal–Wallis test. Bonferroni correction was used to counteract the problem of multiple comparisons. We calculated the Spearman correlation coefficient to quantify the association between negative emotions score and self‐reported overall health. Analyses were conducted using SPSS version 24.0 (IBM Corporation, Armonk, NY, USA). The mediating effect of FT was estimated by the bootstrap method [29] (random sampling with replacement to enlarge the sample, thus making the estimation more accurate) with 5000 samples, performed in IBM SPSS Amos 24.0 (IBM Corporation, Armonk, NY, USA). *p* < 0.05 was considered statistically significant.

### 2.5 Ethical Considerations

This investigation was conducted with approval from the Ethics Committee of Shenzhen People's Hospital (Approval NO. LL‐KY‐2022004‐01) and adhered to the ethical principles outlined in the Declaration of Helsinki. Prior to enrollment, all potential participants received comprehensive information regarding the study objectives and procedures. Written informed consent was obtained from all eligible participants after ensuring their complete understanding of the study protocol. Participants were explicitly informed of their right to withdraw from the study at any time without consequence.

## 3 Results

### 3.1 Descriptive Statistics

The sociodemographic characteristics of the study population are summarized in Table 1. The study cohort had a median age of 51 years (range: 22–87 years), with female participants constituting 59.73% of the sample. The majority of participants (82.10%) resided in urban areas. Regarding employment status, 30.65% were retired, and 19.46% were unemployed. A substantial proportion (44.74%) of participants reported experiencing medical financial burden. The majority of participants (86.58%) were married, while 10.51% reported that their lung cancer diagnosis influenced their planned retirement age. Regarding household monthly income, 8.5% of participants reported earning less than 2000 RMB, 24.16% earned between 2000 and 4999 RMB, 25.50% earned between 5000 and 9999 RMB, and 41.83% reported earning more than 9999 RMB. Nearly half of the participants (48.32%) had attained tertiary education. The majority (63.76%) were enrolled in the Urban Employees Basic Medical Insurance (UEBMI) scheme. Furthermore, three‐quarters of the participants (75.39%) reported having adequate knowledge about their disease.

**TABLE 1:** Descriptive statistics for sociodemographic characteristics and univariate analysis of lung cancer patients (*N* = 447).

Item	*N* (%)	COST score, mean ± SD	*χ* ^2^/*t*/*z*	*p*
Age (years)				
< 40	107 (23.94)	25.43 ± 7.67	3.839	0.147
40–64	258 (57.72)	23.98 ± 8.20		
≥ 65	82 (18.34)	22.90 ± 8.07		
Gender				
Male	180 (40.27)	23.21 ± 8.49	−1.998	0.046
Female	267 (59.73)	24.76 ± 7.74		
Residence				
Urban	367 (82.10)	24.97 ± 7.79	4.798	< 0.001
Rural	80 (17.90)	20.30 ± 8.31		
Employment status				
Employment	223 (49.89)	25.39 ± 7.80	17.220	< 0.001
Unemployment	87 (19.46)	20.55 ± 8.99		
Retired	137 (30.65)	24.36 ± 7.26		
Medical burden level				
No burden at all	70 (15.66)	31.84 ± 6.00	186.243	< 0.001
Basically no burden	177 (39.60)	26.97 ± 6.08		
A certain burden	160 (35.79)	20.09 ± 5.83		
Heavy burden	40 (8.95)	14.23 ± 8.13		
Whether the diagnosis of lung cancer affects retirement age				
No	400 (89.49)	24.79 ± 7.78	5.168	< 0.001
Yes	47 (10.51)	18.53 ± 8.44		
Marital status				
Married	387 (86.58)	24.07 ± 8.10	0.364	0.834
Non‐married	36 (8.05)	25.03 ± 8.59		
Divorced or widowed	24 (5.37)	23.79 ± 7.12		
Household monthly income (RMB Yuan)^a^				
< 2000	38 (8.50)	16.95 ± 9.18	96.091	< 0.001
2000–4999	108 (24.16)	20.48 ± 7.64		
5000–9999	114 (25.50)	23.47 ± 6.25		
> 9999	187 (41.83)	28.10 ± 6.93		
Educational level				
Primary school	41 (9.17)	20.54 ± 8.74	28.381	< 0.001
Middle school	79 (17.67)	21.70 ± 8.16		
High school	111 (24.83)	23.01 ± 8.16		
University or above	216 (48.32)	26.28 ± 7.28		
Medical insurance coverage^b^				
UEBMI	285 (63.76)	25.27 ± 7.57	34.353	< 0.001
URBMI	96 (21.48)	24.32 ± 8.10		
NRCMS	54 (12.08)	17.67 ± 8.04		
Others	12 (2.68)	24.75 ± 7.00		
Smoking				
No	342 (76.51)	24.70 ± 7.88	2.725	0.007
Yes	105 (23.49)	22.27 ± 8.46		
Knowledge of the disease				
Understanding	337 (75.39)	25.05 ± 7.78	17.228	< 0.001
General acquaintance	69 (15.44)	22.35 ± 7.98		
No knowledge	41 (9.17)	19.56 ± 8.71		

^a^ Household monthly income (RMB Yuan): classified as low, middle, higher middle, and high income for < 2000 (RMB Yuan), 2000–4999, 5000–9999, and > 9999.

^b^ Medical insurance coverage: The Urban Employees Basic Medical Insurance (UEBMI) covering 80%–90%; The Urban Residents Basic Medical Insurance (URBMI) covering 70%–80%; The New Rural Cooperatives Medical Scheme (NRCMS) covering 50%–60% of the medical expenses.

Clinical characteristics of the study population, including disease and treatment parameters, are summarized in Table 2. Pathologic staging revealed that the majority of participants (73.38%) presented with Stage I disease. Adenocarcinoma was the predominant histological subtype, accounting for 58.84% of cases. A small proportion of patients (6.04%) had received chemotherapy. With respect to disease chronicity, approximately half of the cohort (50.11%) reported a cancer duration of 1–4 years.

**TABLE 2:** Descriptive statistics for clinical (disease and treatment) profile and univariate analysis of lung cancer patients (*N* = 447).

Item	*N* (%)	COST score, mean ± SD	*χ* ^2^/*t*/*z*	*p*
Tumor staging^a^				
I	328 (73.38)	25.65 ± 7.52	41.609	< 0.001
II	79 (17.67)	20.56 ± 7.50		
≥ III	28 (6.26)	19.39 ± 9.00		
Not sure	12 (2.68)	21.92 ± 9.27		
Pathological pattern				
Adenocarcinoma	263 (58.84)	25.18 ± 7.60	15.334	0.002
Squamous carcinoma	92 (20.58)	23.90 ± 7.99		
Small‐cell carcinoma	77 (17.23)	21.23 ± 8.96		
Other	15 (3.36)	22.07 ± 8.59		
Chemotherapy				
No	420 (93.96)	24.38 ± 7.96	2.612	0.009
Yes	27 (6.04)	20.22 ± 9.03		
Duration of cancer				
< 1 year	165 (36.91)	23.96 ± 8.05	3.653	0.161
1–4 year	224 (50.11)	23.79 ± 7.97		
> 4 year	58 (12.98)	25.93 ± 8.46		

^a^ Tumor staging based on the American Society of Cancer Association 8th Edition TNM staging criteria.

Financial toxicity, as measured by the COST‐PROM instrument, yielded a median score of 22 (range: 1–44) in the study population. When stratified by tertiles, scores in the lowest tertile ranged from 1 to 22, while the combined middle and upper tertiles encompassed scores from 23 to 44. Regarding psychological status, the NESSI assessment revealed a mean score of 33.42 (SD = 11.99; range: 17–85) among participants.

Significant associations were observed between FT and multiple sociodemographic and clinical variables among lung cancer patients. These variables included age, gender, residential location, employment status, impact of diagnosis on retirement timing, monthly household income, educational attainment, type of medical insurance coverage, smoking status, disease‐related knowledge, tumor stage, and pathological subtype (all *p* < 0.05).

### 3.2 Correlations Between Perceived Social Support, Financial Toxicity, and Negative Emotions

Pearson's correlation analyses (Table 3) revealed several significant associations among the key study variables. Perceived social support demonstrated a weak negative correlation with negative emotions (*r* = −0.13, *p* < 0.05), indicating that patients with higher levels of perceived social support (higher PSSS scores) experienced fewer negative emotions. A moderate negative correlation was observed between FT scores and negative emotions (*r* = −0.50, *p* < 0.01), suggesting that greater financial burden was associated with increased negative emotional states. Additionally, perceived social support exhibited a positive correlation with FT score (*r* = 0.26, *p* < 0.01), representing a moderate effect size, elaborating that the higher the perceived social support, the lower the levels of FT.

**TABLE 3:** Correlation matrix between negative emotion, social support, and financial toxicity.

	M	SD	1	2	3
1. Negative emotion	33.42	11.99	1.00		
2. Social support	63.97	12.65	−0.13*	1.00	
3. Financial toxicity	24.13	8.08	−0.50**	0.26**	1.00

* *p* < 0.05.

** *p* < 0.01.

### 3.3 Model Test

**FIGURE 1:**
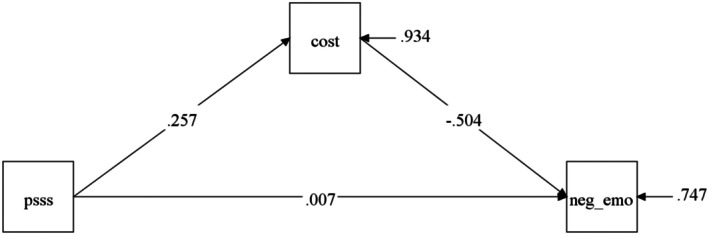
The mediating effect of social support affecting negative emotion through financial toxicity.

The interrelationships among social support, FT, and negative emotions were examined using structural equation modeling (SEM). The model incorporated relevant covariates, including employment status, medical burden level, impact of diagnosis on retirement timing, household monthly income, educational attainment, medical insurance coverage, disease‐related knowledge, and tumor stage (Figure 1). The SEM analysis revealed that FT significantly predicted negative emotions (*β* = −0.504, *p* < 0.01). Together, social support and FT accounted for 25.3% of the variance in patients' negative emotions. Social support demonstrated a significant positive association with FT (*β* = 0.257, *p* < 0.01), explaining 6.60% of its variance. Notably, FT emerged as a significant mediator in the relationship between social support and negative emotions (indirect effect = −0.124, 95% CI: −0.182 to −0.086; Table 4).

**TABLE 4:** Mediated model effect test of social support affecting negative emotion through financial toxicity.

Social support → negative emotion	Effects	95% bootstrap CI	Proportion of mediating effect
Total effect	−0.118*	(−0.226, −0.015)	
Social support → financial toxicity→ negative emotion	−0.124***	(−0.182, −0.086)	95%
Direct effect	0.006	(−0.094, 0.102)	

* p < 0.05.

*** *p* < 0.001.

## 4 Discussion

To our knowledge, this investigation represents the first comprehensive examination of the interrelationships among perceived social support, FT, and negative emotions in post‐surgical lung cancer patients. The concept of FT remains understudied in the Chinese context, particularly among surgically treated lung cancer patients. Financial burden in this population is multi‐factorial, influenced by income levels, socioeconomic status, treatment‐associated costs, and disease burden. Despite the study's setting in a relatively affluent region, participants reported substantial levels of FT. As hypothesized, post‐surgical lung cancer patients demonstrated elevated FT scores, with a considerable proportion (42.51%) experiencing severe financial distress. These findings align with previous research [30] that documented similar prevalence rates of FT. However, the observed FT prevalence in our cohort was comparatively lower than rates reported in Chinese colorectal cancer patients (52.8%) [31] and American gynecologic malignancy patients (58%) [32]. Recent data from the National Health Interview Survey (NHIS) in the United States indicated that 55% of surgical patients experienced FT [33]. The variability in FT prevalence across studies may be attributed to methodological heterogeneity, including differences in study design, cancer types, participant characteristics, sociodemographic factors, and FT assessment tools [34]. Therefore, our findings specifically illuminate the financial challenges faced by surgically treated lung cancer patients within a high‐middle income region of China, where universal healthcare coverage is provided through public healthcare facilities.

### 4.1 Sociodemographic/Clinical Variables and Patients' Financial Toxicity

Analysis of sociodemographic and clinical characteristics revealed several significant predictors of FT among lung cancer patients. Higher FT was significantly associated with male gender, rural residence, unemployment status, substantial medical burden, impact of diagnosis on retirement timing, lower household monthly income, lower educational attainment, inadequate medical insurance coverage or self‐payment, smoking history, limited disease‐related knowledge, advanced disease stage, and chemotherapy treatment. Consistent with Liu et al. [35], male participants demonstrated higher levels of FT compared to females. This gender disparity may be attributed to traditional social roles where men, as primary household breadwinners, experience greater financial strain due to illness‐related productivity losses and associated psychological stress. While our analysis showed no significant age‐related differences in FT, contrary to findings by Mo et al. [31], who reported increased financial hardship among younger patients [31]. Educational attainment demonstrated an inverse relationship with FT, corroborating findings by Thaduri et al. [36]. Furthermore, aligned with previous research [37], our results indicated that rural residents and those covered by Urban Employee Basic Medical Insurance were particularly vulnerable to experiencing elevated FT. China's cancer care system is primarily anchored by the Basic Medical Insurance (BMI) scheme, which provides coverage to approximately 95% of the population [38]. This universal coverage is achieved through three government‐established social health insurance programs: Urban Employee Basic Medical Insurance (UEBMI), Urban Resident Basic Medical Insurance (URBMI), and the New Rural Cooperative Medical Scheme (NRCMS). The UEBMI is jointly financed by employers and employees. It primarily covers urban employees and retirees within the formal sector, notably including individuals who previously benefited from free medical care in public institutions and state‐owned enterprises. The NRCMS provides coverage for rural residents, while the URBMI covers non‐UEBMI‐eligible urban populations, including unemployed individuals and children. Among these three types, UEBMI demonstrates the highest reimbursement rates, followed by URBMI and NRCMS [39]. Furthermore, BMI also does not comprehensively cover the costs associated with innovative therapies or advanced diagnostic technologies, thereby exposing patients to substantial out‐of‐pocket expenditures. Although public‐private insurance models are under exploration, commercial health insurance remains underutilized, accounting for a mere 3.8% of total health expenditure [40]; this limited penetration constrains patients' financial protection during the often prolonged course of cancer treatment. Therefore, to help mitigate FT risk in households with lung cancer patients, we suggest that the central government adjust the related policies by types of diseases and medical insurance reimbursement rates.

Analysis of clinical characteristics revealed a significant association between disease stage and FT, with advanced‐stage lung cancer patients experiencing heightened levels of FT compared to those with early‐stage disease. This relationship may be attributed to the deteriorating health status associated with advanced disease, necessitating more complex therapeutic interventions and consequently increasing treatment‐related expenditures. Moreover, the complications accompanying advanced disease often impede patients' ability to maintain employment, further exacerbating their financial burden [35]. Our analysis demonstrated that patients undergoing chemotherapy experienced significantly higher FT compared to those receiving other treatment modalities, corroborating findings from previous investigations by Thaduri et al. [36] and Mo et al. [31]. The elevated FT in this subgroup can be primarily attributed to the substantial costs associated with chemotherapeutic interventions. Despite ongoing initiatives by the Chinese government to enhance medical insurance coverage for antineoplastic medications and establish comprehensive healthcare security systems, patients continue to face considerable financial hardship due to substantial treatment‐related expenses [31]. These findings underscore the necessity for targeted policy interventions aimed at reducing treatment‐related expenditures for lung cancer patients. Such measures could contribute significantly to alleviating FT while facilitating more efficient allocation of limited healthcare resources. In recent years, financial navigation (FN) has been proposed as the most frequently used approach and was increasingly acknowledged as a promising strategy to mitigate FT among cancer survivors [41]. Evidence has confirmed its effectiveness by systematically identifying patients at high risk for FT across the survivorship journey, initiating cost‐related conversations, estimating treatment costs, providing personalized out‐of‐pocket cost information and guidance aligned with individual financial circumstances, facilitating access to assistance programs and developing practical expense management strategies to address financial hardship [42]. A case series suggested that engaging with FN could help cancer patients save an average of $33,265 annually from free medications, $35,293 from premium assistance, $12,256 through insurance optimization, and $3076 from co‐pay assistance [43]. On the other hand, mobile health (mHealth) technologies demonstrate significant potential for enhancing service delivery and intervention implementation in cancer care. Recent studies found that interventions with remote delivery (e.g., mobile applications, web‐based platforms, videoconferencing and machine learning) could achieve higher feasibility and retention [44, 45].

A significant finding of our investigation was the strong association between FT and several socioeconomic factors, including employment status, medical burden, impact of diagnosis on retirement timing, and household monthly income. These associations align with previous research on cancer patients [31]. Evidence suggests that individuals who experience job loss or substantial medical burdens typically have reduced savings and limited access to stable welfare benefits. Premature retirement or unemployment frequently leads to diminished quality of life and economic instability. Previous studies have demonstrated that cancer diagnosis and subsequent treatment often result in reduced job retention and fewer working hours, consequently limiting patients' ability to accumulate assets and maintain stable income [31]. Moreover, the psychological burden and disease‐related distress associated with prolonged treatment regimens can significantly impair individuals' work capacity and long‐term productivity [46]. The impact extends beyond the patient to family caregivers, who often must dedicate substantial time and resources to caregiving responsibilities, resulting in reduced personal income [47]. These compounding factors collectively contribute to decreased household income and severe financial hardship. The implications of significant FT are multifaceted, predominantly affecting patients' social and environmental quality of life dimensions, particularly their capacity to maintain employment and work productivity. This creates a self‐perpetuating cycle where financial hardship and diminished work capacity mutually reinforce each other, potentially leading to long‐term socioeconomic consequences. Research suggests that cancer patients' reintegration into society through workforce participation not only restores economic productivity but also mitigates long‐term FT [48]. For cancer patients who are unable to resume formal employment or are beyond conventional working age, engagement in unpaid productive activities—including household management and familial caregiving—can generate compensatory economic value that alleviates household financial burden [49].

### 4.2 Perceived Social Support, Financial Toxicity, and Negative Emotions

A particularly noteworthy finding of our investigation was the negative correlation between perceived social support and negative emotions, with FT serving as a mediating factor. Our analysis revealed that social support exerted indirect effects on psychological outcomes among post‐surgical lung cancer patients. While social support traditionally serves as a protective factor against psychological distress, our findings suggest that its relationship with emotional well‐being is more complex than previously understood. Previous research has demonstrated that robust social support systems can effectively reduce negative emotions, including anxiety and depression [31]. Caregivers' practical and material support can contribute significantly to alleviating the overall financial burden. Psychological distress, encompassing anxiety, depression, and sadness related to cancer diagnosis and treatment, represents a crucial component of negative emotions. Patients who undergo extensive surgical resection, requiring multi‐modal treatment approaches, often experience bodily disfigurement and occupational changes, potentially increasing their susceptibility to negative emotions [36]. Our results demonstrated a negative association between FT and emotional well‐being, indicating that higher levels of FT correspond with increased emotional distress. This finding aligns with existing literature documenting significant correlations between FT and negative psychological outcomes [36]. Notably, Kale and Carroll [50] reported that cancer patients experiencing economic burden had 1.95 times higher odds of developing depression compared to those without financial constraints, with odds increasing proportionally to financial burden severity. The interplay between FT and psychological distress can impose a substantial burden on cancer care, both for survivors and patients undergoing treatment. Increased FT and poor psychological outcomes may significantly affect treatment adherence and overall quality of life. Understanding the financial challenges faced by patients could inform treatment decisions and improve patient‐centered care. Strengthening communication between oncologists, nurses, and patients is therefore essential. Open discussions about medical expenditures and collaboratively selecting treatments tailored to patients' financial and personal circumstances are critical to ensuring high‐quality care and preserving patients' quality of life. Another study's findings suggested that the cost of care discussion was beneficial to relieve patients' financial concerns about cancer treatment [51]; however, 75% and 80% of participants still did not have a clear understanding of the estimation of out‐of‐pocket costs and options for eligible financial aid programs, respectively, which indicated the need for intensive patient‐provider financial discussions [45]. Furthermore, lung cancer patients can easily access financial counselors who could provide practical advice and guidance to help manage financial hardship induced by the disease [42].

## 5 Limitations

Several methodological limitations warrant consideration when interpreting and generalizing these findings. First, the single‐center design, focusing exclusively on post‐surgical lung cancer patients, limits the generalizability of results to other clinical settings or treatment modalities. Additionally, the study was conducted in a region with significantly higher economic development than the national average, potentially introducing socioeconomic bias. Future research would benefit from multi‐center designs incorporating larger sample sizes and diverse geographic regions with varying economic profiles to validate our findings. Second, the cross‐sectional nature of this investigation provides only a snapshot of the relationships among variables at a single time point, precluding causal inference. To address this limitation, longitudinal studies and prospective controlled trials are necessary to elucidate the temporal dynamics of FT throughout the cancer trajectory and provide more comprehensive insights into the financial experiences of lung cancer survivors. Third, this study's reliance on self‐reported measures (PSSS, COST‐PROM, and NESSI) makes these instruments susceptible to recall bias, social desirability bias, and potential interpretation differences among participants. The absence of objective measures for both social support and financial status limits our ability to validate subjective perceptions against external benchmarks. These limitations highlight the need for future research to incorporate mixed‐methods designs or objective indicators, thereby mitigating measurement biases, strengthening causal inference, and enhancing the understanding of FT in oncology populations to inform evidence‐based interventions.

## 6 Clinical Implications

This investigation offers several notable strengths. First, it represents a novel examination of FT among post‐surgical lung cancer patients, incorporating a relatively large sample size compared to previous studies. The research utilized well‐established, validated instruments to assess social support, FT, and negative emotions, enhancing the reliability of our findings. Notably, the COST measure employed in this study has been specifically validated for cancer populations [13], lending additional methodological rigor to our assessment of FT. Our findings revealed elevated levels of FT among post‐surgical lung cancer patients and demonstrated complex interrelationships among key variables. Specifically, perceived social support demonstrated a protective effect against negative emotions, while FT exhibited an amplifying effect on emotional distress. Furthermore, we identified a significant positive association between perceived social support and patients' experience of FT. These findings have important implications for clinical practice. Healthcare professionals may leverage these insights to develop targeted interventions that enhance social support mechanisms and address financial concerns among lung cancer patients. Future research should focus on developing and evaluating intervention programs designed to mitigate FT and negative emotions through enhanced social support frameworks.

## 7 Conclusion

This investigation makes significant contributions to the expanding literature on FT among lung cancer patients. Despite the study population's relatively high socioeconomic status and comprehensive insurance coverage, our findings demonstrate that FT remains a substantial burden for post‐surgical lung cancer patients, even within a high‐middle income region of China. A particularly noteworthy finding is the mediating role of FT in the relationship between perceived social support and negative emotions. This complex interplay underscores the importance of early identification and intervention for both financial hardship and psychological distress among cancer patients. These findings have important implications for healthcare policy and clinical practice. There is an urgent need for medical institutions and governmental bodies to implement effective interventions addressing FT in cancer care. Such initiatives are crucial for mitigating the long‐term impact of financial burden on cancer survival outcomes and patients' quality of life. Our results emphasize the necessity of developing comprehensive support systems that address both the financial and psychological needs of lung cancer patients. Future healthcare policies should focus on creating sustainable solutions to reduce financial burden while enhancing psychosocial support mechanisms for this vulnerable population.

## Author Contributions

**Julan Xiao:** writing – original draft, data curation, writing – review and editing, investigation, resources, conceptualization, methodology, project administration. **Lili Liu:** writing – review and editing, methodology, data curation, conceptualization. **Yanheng Xie:** writing – review and editing, investigation. **Sijiao Cheng:** writing – review and editing, investigation. **Binghu Lin:** writing – review and editing, investigation. **Lansong Qin:** writing – review and editing, investigation. **Fengjiao Chen:** writing – review and editing, investigation. **Huai An:** investigation, writing – review and editing. **Wanling Xu:** writing – review and editing, investigation. **Yi Wen:** writing – review and editing, investigation, methodology, supervision, resources. **Weixiang Luo:** writing – review and editing, methodology, conceptualization, supervision, project administration.

## Conflicts of Interest

The authors declare no conflicts of interest.

## Data Availability

The datasets that support the findings of the current study are available from the corresponding author on reasonable request.
